# Human monoclonal antibodies inhibit invasion of transgenic *Plasmodium knowlesi* expressing *Plasmodium vivax* Duffy binding protein

**DOI:** 10.1186/s12936-023-04766-1

**Published:** 2023-12-04

**Authors:** Quentin D. Watson, Lenore L. Carias, Alyssa Malachin, Karli R. Redinger, Jürgen Bosch, Martino Bardelli, Lea Baldor, Lionel Brice Feufack-Donfack, Jean Popovici, Robert W. Moon, Simon J. Draper, Peter A. Zimmerman, Christopher L. King

**Affiliations:** 1 Center for Global Health and Diseases, Case Western Reserve University School of Medicine, Cleveland, OH USA; 2 Department of Biochemistry, University of Oxford, Oxford, UK; 3 Malaria Research Unit, Institut Pasteur du Cambodge, Phnom Penh, Cambodia; 4 Faculty of Infectious and Tropical Diseases, London School of Hygiene and Tropical Medicine, London, UK; 5 Veterans Affairs Medical Center, Cleveland, OH USA

**Keywords:** A monoclonal antibody, *Plasmodium vivax*, *Plasmodium knowlesi*, Duffy binding protein, Duffy antigen

## Abstract

**Background:**

*Plasmodium vivax* has been more resistant to various control measures than *Plasmodium falciparum* malaria because of its greater transmissibility and ability to produce latent parasite forms. Therefore, developing *P. vivax* vaccines and therapeutic monoclonal antibodies (humAbs) remains a high priority. The Duffy antigen receptor for chemokines (DARC) expressed on erythrocytes is central to *P. vivax* invasion of reticulocytes. *P. vivax* expresses a Duffy binding protein (PvDBP) on merozoites, a DARC ligand, and the DARC: PvDBP interaction is critical for *P. vivax* blood stage malaria. Therefore, PvDBP is a leading vaccine candidate for *P. vivax* and a target for therapeutic human monoclonal antibodies (humAbs).

**Methods:**

Here, the functional activity of humAbs derived from naturally exposed and vaccinated individuals are compared for the first time using easily cultured *Plasmodium knowlesi* (*P. knowlesi*) that had been genetically modified to replace its endogenous PkDBP orthologue with PvDBP to create a transgenic parasite, PkPvDBPOR. This transgenic parasite requires DARC to invade human erythrocytes but is not reticulocyte restricted. This model was used to evaluate the invasion inhibition potential of 12 humAbs (9 naturally acquired; 3 vaccine-induced) targeting PvDBP individually and in combinations using growth inhibition assays (GIAs).

**Results:**

The PvDBP-specific humAbs demonstrated 70–100% inhibition of PkPvDBPOR invasion with the IC_50_ values ranging from 51 to 338 µg/mL for the 9 naturally acquired (NA) humAbs and 33 to 99 µg/ml for the 3 vaccine-induced (VI) humAbs. To evaluate antagonistic, additive, or synergistic effects, six pairwise combinations were performed using select humAbs. Of these combinations tested, one NA/NA (099100/094083) combination demonstrated relatively strong additive inhibition between 10 and 100 µg/mL; all combinations of NA and VI humAbs showed additive inhibition at concentrations below 25 µg/mL and antagonism at higher concentrations. None of the humAb combinations showed synergy. Invasion inhibition efficacy by some mAbs shown with PkPvDBPOR was closely replicated using *P. vivax* clinical isolates.

**Conclusion:**

The PkPvDBPOR transgenic model is a robust surrogate of *P. vivax* to assess invasion and growth inhibition of human monoclonal Abs recognizing PvDBP individually and in combination. There was no synergistic interaction for growth inhibition with the humAbs tested here that target different epitopes or subdomains of PvDBP, suggesting little benefit in clinical trials using combinations of these humAbs.

## Background

The estimated annual global burden of *Plasmodium vivax* malaria is 14.3 million (13.7 to 15.0 million) cases [1]. However, this approximation of *P. vivax* clinical cases grossly underestimates *P. vivax* asymptomatic or latent infections in the liver, leading to more subtle morbidity and death in impoverished settings where endemic populations frequently experience malnutrition, co-infections, and limited access to health care [1–3].

*Plasmodium vivax* infections can also include Duffy-negative individuals in sub-Saharan Africa, previously considered protected from *P. vivax* erythrocytic invasion [4–6]. Although there has been a steady decrease in the malaria burden, particularly for *P. falciparum*, in the last decade, the impact is much less pronounced for *P. vivax* because of latent infections and greater transmissibility in *P. vivax* endemic areas [4]. In addition, this trend has stagnated recently because of political and economic instability and the global health crisis caused by the COVID-19 pandemic [7–9]. To address the burden of *P. vivax* malaria, additional strategies are needed.

*Plasmodium vivax* initiates blood-stage infections by invading immature red blood cells (RBCs) or reticulocytes using its endogenous Duffy binding protein (PvDBP) to access the Duffy antigen receptor for chemokines (DARC) (encoded by gene atypical chemokine receptor 1, ACKR1 [10–13]. The structural biology for the PvDBP and DARC interaction has become increasingly well defined [14–18]. Among six distinct structural regions, the cysteine-rich region II (PvDBPII) contains three subdomains (SD) [12]. SD2 contains the Duffy antigen binding motif [10]. As PvDBPII is the most polymorphic region, it is suggested to be under strong selection pressure [19, 20]. This motif interacts with DARC's N-terminal 30 amino acid region to form the heterotetramer necessary for the binding interaction and commitment to the subsequent invasion [15]. The necessity of PvDBPII for *P. vivax* invasion of reticulocytes makes it a primary target for host immunity. Evidence supporting this hypothesis has included observations of extensive amino acid variation (most highly abundant in SD2 and SD3) [21–23] and recent Phase I/IIa vaccine efficacy of PvDBPII against blood-stage *P. vivax* infection [24].

Previous researchers have produced murine monoclonal antibodies to PvDBPII that blocked the binding of DBPII to DARC in various binding inhibitory assays [25, 26]. However, they were not strain-transcending and failed to inhibit *P. vivax* invasion of reticulocytes in vitro [25–27]. These murine mAbs recognized SD3 of PvDBPII, which does not contain the binding motif to DARC. Still, SD3 is important for developing the heterotetramer necessary for stable binding interaction to DARC [14, 15]. In comparison, it has been previously identified 9 to 15% of individuals living in *P. vivax* endemic areas of Papua New Guinea, Cambodia, and Brazil with antibodies to PvDBPII capable of blocking PvDBPII from binding to DARC and preventing *P. vivax* invasion of reticulocytes [28–33]. High levels of these blocking antibodies correlate with reduced risk of infection and disease in human cohort studies [28–33]. From some individuals with binding inhibitory antibodies to PvDBPII, PvDBPII-specific memory B cells were isolated to generate a panel of human monoclonal antibodies (humAbs) [33]. Three of these humAbs have been tested in a short-term ex vivo growth assay using clinical *P. vivax* isolates from Cambodia and Brazil. Notably, these humAbs exhibited strain-transcending inhibition of *P. vivax* reticulocyte invasion by up to 80% at 100 µg/mL, and two of these humAbs recognized the predicted DARC binding site in PvDBPII SD2 [22, 27, 33].

Additional humAbs have been generated from healthy volunteers immunized with a vaccinia virus vectored vaccine expressing Salvador I strain (Sal I) PvDBPII in a Phase Ia clinical vaccine trial [34]. The humAbs from this vaccine trial were validated in recombinant PvDBP-DARC binding inhibition assays, *ex-vivo P. vivax* invasion assays, and *P. knowlesi* growth inhibition assays using the *P. knowlesi* strain A1-H.1 PvDBP OR /Δ14 (PkPvDBPOR), which has been CRISPR-Cas9 modified to replace *P. knowlesi*’s endogenous DARC binding protein with PvDBP and adapted to grow in continuous human culture [35–38]. Several of these humAbs displayed strain-transcendent blocking of recombinant PvDBPII to the DARC ectodomain and inhibited invasion, including an SD3-specific humAb [38]. To build upon this research the PkPvDBPOR in vitro model system was used to examine inhibition of human erythrocyte invasion by naturally acquired PvDBPII-specific humAbs [33]. The analysis of vaccine-induced and naturally acquired humAbs was expanded, individually and in combination, to identify potential additive and/or synergistic effects.

## Methods

### Human blood preparation

Venous blood was collected from healthy consented donors in EDTA vacutainers. The blood was centrifuged (1500×*g* for 5 min) to separate plasma and cellular material. Plasma was aspirated, and the remaining blood was passed through a neonatal blood filter (Haemonetics NEO1) for leukocyte depletion, washed with PBS, centrifuged (2000×*g* for 8 min), and the resulting supernatant removed. An equal volume of *P. knowlesi* complete medium (see below) is added to bring the blood to 50% hematocrit and stored at 4 ºC. Stored blood will support culture growth for approximately 3 weeks. Fresh blood is acquired every 2 weeks or earlier. The Duffy (Fy) genotype was assessed as previously described [39]. Donors for *P. knowlesi* culture were either Fy A + /B + or Fy B + /B + .

### Monoclonal antibodies

Cloning, expression, and purification of nine human PvDBP-specific monoclonal antibodies (humAbs: 099100; 080086; 055056; 071063; 053054; 092096; 065098; 081082; 094083) have been previously described [33, 40, 41]. Three PvDBP-specific humAbs (DB9; DB10; DB42) were generated from individuals exposed to a vaccinia virus vectored vaccine expressing Salvador I strain (Sal I) PvDBPII in a Phase Ia clinical vaccine trial [34]. A humAb specific for tetanus toxoid C-terminal (043048) was used as a negative control. Protein concentration was determined using a Nanodrop at 280 nm. The mAbs were concentrated to > 4 mg/ml and filter-sterilized through a 0.22 µm PVDF filter for subsequent use. As a control, the nanobody CA111 was used to demonstrate invasion inhibition of PkPvDBPOR. CA111 is specific to the Fy6 epitope on DARC that blocks the binding of PvDBP to DARC as previously described [42].

### *Plasmodium knowlesi *in vitro culture

*Plasmodium knowlesi* culture media (PkCM) included RPMI 1640 medium (22400, Gibco) supplemented with 1.15 g/L sodium bicarbonate, 1 g/L dextrose, 0.05 g/L hypoxanthine, 5 g/L Albumax II, 0.025 g/L gentamicin sulfate, 0.292 g/L L-glutamine, and 10% (vol/vol) heat-inactivated horse serum (26050, Gibco) referred to as PkCM as previously described [37]. *Plasmodium knowlesi* cultures were maintained in sealed flasks with 5% O_2_, 5–7% CO_2_, balanced by nitrogen.

Cryopreserved isolates of *P. knowlesi* A1.H.1 strain (1 mL) were thawed by drop-wise addition of 3.5% (weight/vol) NaCl over 1 min, then transferred to a 15 ml Falcon tube. Thawed parasites were centrifuged (1500xg, 5 min), and the supernatant was discarded. This treatment was repeated with 3.5% NaCl three times. The treated parasites were pelleted (1500×*g* for 5 min) and resuspended in 1 mL of warm PkCM. This resuspension was added to 50 mL PkCM plus 1 mL of fresh RBCs (2% Haematocrit).

The parasitaemia of routine cultures was maintained below 5% and expanded to 8–12% for growth inhibition assays. Culture maintenance included medium changes/dilution of parasites every 2–3 days. Parasite viability was monitored by Giemsa-stained culture smears made during culture changes. *Plasmodium knowlesi* cultures were expanded for at least 4 life cycles, and synchronized using Nycodenz gradient to enrich schizonts for growth inhibition assays.

### Nycodenz synchronization

Parasites were synchronized using Nycodenz (157,750, MP Biomedicals) as described previously [37]. Nycodenz is a non-ionic tri-iodinated derivative of benzoic acid [43]. Nycodenz stock solution is prepared at 27.6% (weight/vol) Nycodenz, 10% (vol/vol) 100 mM HEPES (BP299, Fisher BioReagents), adjusted to pH 7.0, supplemented with sterile distilled H_2_O to reach final concentrations, and then filter sterilized. A Nycodenz working solution (55% vol/vol of stock solution) comprises 55 mL of Nycodenz stock solution to 45 mL of PkCM (without serum). Parasite cultures were centrifuged (1500xg, 8 min). The supernatant is aspirated, and the pellet is resuspended in 1 mL PkCM, to approximately 50% hematocrit, for a total volume of 2 mL. Parasites were layered over 5 mL Nycodenz working solution in a 15 mL conical tube and centrifuged (900xg, 12 min) with low brake/acceleration. The brown interphase containing schizonts is pipetted off and pelletized in a microcentrifuge (1000xg, 1 min). Pellet is washed once in 1 mL of PkCM, and once in 1 × PBS before CellTrace staining. Immediately following Nycodenz enrichment, direct blood smears demonstrated 50–60% schizonts and 1–2% ring stage or trophozoites.

### Growth inhibition assays (GIAs)

The Nycodenz-enriched schizonts (donor cells) were stained in CellTrace Far-red (C34564, Invitrogen) at 4.5 µM in 1 × PBS for 30 min at 37 ℃ on a rotator in the dark. Donor cells were centrifuged (1000xg, 1 min), the supernatant aspirated, and the pellet resuspended in 5 mL of PkCM for the growth inhibition assay. PkCM (50 µl) with humAbs were first aliquoted in 96 well flat bottomed microwell plates. To reach a final culture volume of 100 µl cultures, 25 µl of 8% RBCs (unlabelled recipients) in PkCM (final haematocrit of 2%) was added, followed by 25 µl of donor cells for a 1:20 ratio (donor: recipient). An additional well was made for the 0 h time point, parasites were removed and fixed to establish parasitaemia and time zero invasion events. The remaining enrichment preparation was fixed to verify CellTrace labelling efficiency by flow cytometry. The 96 well plates were placed in a modular incubator chamber (MIC-101), gas for 2 min (5% O_2_, 7% CO_2_, balanced by nitrogen), sealed, and incubated in a 37℃ incubator for 6 h. At the end of the culture period, experimental samples were fixed in a 1 × PBS solution containing 4% paraformaldehyde and 0.01% glutaraldehyde for 20 min at room temperature. Cells were centrifuged (1000×*g*, 5 min) and washed once with 1 × PBS. Cells were stored in 1 × PBS at 4 °C or immediately stained for subsequent flow cytometry.

### Flow cytometric evaluation of GIAs

Samples were stained in a 1 × PBS solution containing Hoechst 33,342 at 4 µM for DNA content (parasites) and thiazole orange at 100 ng/mL, for preferential staining of RNA and reticulate matter in reticulocytes, for a minimum of 30–40 min at room temperature, or overnight at 4 ℃. Samples are then monitored and analysed by flow cytometry (Biosciences BD LSR II Flow Cytometer). The Hoechst dye was excited by the ultraviolet 355 nm laser (excitation peak 355 nm, emission peak 465 nm) and detected using a 440/40 filter. Thiazole orange was excited by a blue 488 nm laser (excitation peak 514 nm, emission peak 533 nm) and detected using a 525/20 filter. CellTrace Far-red was excited by the red 640 nm laser (excitation peak 630 nm, emission peak 661 nm) and detected using the R660/20 filter. The resulting FCS files were analyzed by Flowjo 10.8.1 software for growth inhibition. The percent growth inhibition was calculated by comparing invasion events in the CellTrace negative Ring gate at 6 h divided by 6 h control invasion events, minus zero-hour events.

### In vitro invasion inhibition assay using *Plasmodium vivax* clinical isolates

Clinical isolates of *P. vivax* were cryopreserved, and later thawed and cultured in IMDM medium (Gibco) supplemented with 0.5% Albumax II (Gibco), 2.5% heat-inactivated human serum, 25 mM HEPES (Gibco), 20 µg/mL gentamicin (Sigma) and 0.2 mM hypoxanthine (C–C Pro) for ~ 24 or ~ 48 h until a majority of schizont stage parasites were observed as previously described [44]. The schizont-infected erythrocytes were enriched using KCl-Percoll density gradient [45], then added to uninfected RBCs. Uninfected RBCs were prepared at a ratio of 1:1 (mature erythrocytes: reticulocyte enriched from cord blood) and labelled with CellTrace Far-red dye following the manufacturer’s instructions. The mixed samples were incubated for ~ 8 h in a final volume of 50 μL in 96 well plates or 20 μL in 384 well plates in the presence of the humAbs. Medium alone was used as a control for invasion normalization, and the mouse monoclonal anti-Duffy 2C3 at 100 µg/mL was used as positive invasion inhibition control. Post-invasion, cells were stained with Hoechst 33342 and examined by flow cytometry. Reticulocytes which were Hoechst 33342 and Far-Red positive, were scored as new invasion events.

### Avidity assays

96-well Immulon 4 HB plates were coated with 0.5 µg/mL recombinant PvDBPII overnight at 4 ℃, washed, and blocked with 3% BSA in 1 × PBS for 1-2 h at 37 ℃. Plates were washed and incubated with 0.5 µg/mL humAbs at 50 µl/well in duplicate, and incubated for 1 h at 37 ℃. After washing, wells were treated with NH_4_SCN at 0.25, 0.5, 0.75, and 1 M for 15 min at room temperature. Control wells are treated with PBS. After washing, plates were incubated with 1/1000 dilution of HRP conjugated to anti-human IgG (Fc) (BD Pharminigen) for 1 h at 37 ℃ followed by TMB Peroxidase EIA Substrate (Bio-Rad), and the reaction was stopped with 10% Sulfuric acid. Colormetric reading was performed by VersaMax Tunable Microplate Reader. Data is processed in SoftMax Pro 6.2.1.

### Statistical analysis

A nonlinear regression curve analysis was applied to estimate the IC_50_ (antibody potency) and R^2^ values using GraphPad Prism 9 software. The Synergy Finder 2.0 web-based application software evaluated the synergistic, additive, or antagonistic effects of a combination of two humAbs based on the independent model Bliss [46]. Synergy Finder generates a 3-dimensional representation of the dose–response matrices showing the concentrations of one humAb on the x-axis, a second humAb on the y-axis, and the synergy score (δ) on the z-axis; each matrix is colour coded to show synergy distribution, a corresponding Bliss synergy score (BSS), and topography (peaks/valleys) at specific concentrations; green/valleys denote antagonism, red/peaks denote additivity or synergy. Scale for BSS: score < 0 = antagonism, 10 < score > 0 = additivity, score > 9.9 = synergy.

## Results

### Characteristics of individual humAb in the PkPvDBPOR growth inhibition assay (GIA)

To examine and compare the functional activity of the NA and VI humAbs, a modified GIA was developed with PkPvDBPOR. The assay requires the addition of CellTrace-labelled, enriched parasitized cells (donor) to uninfected cells (recipient). The plasma membranes of donor cells are labelled with CellTrace to ensure identification of newly invaded recipient target cells (CellTrace negative) instead of identification of infected donor cells from the routine culture. This experimental design optimized the detection of new erythrocyte invasion events and was the basis of the assessment of humAb inhibition characteristics.

**Fig. 1:**
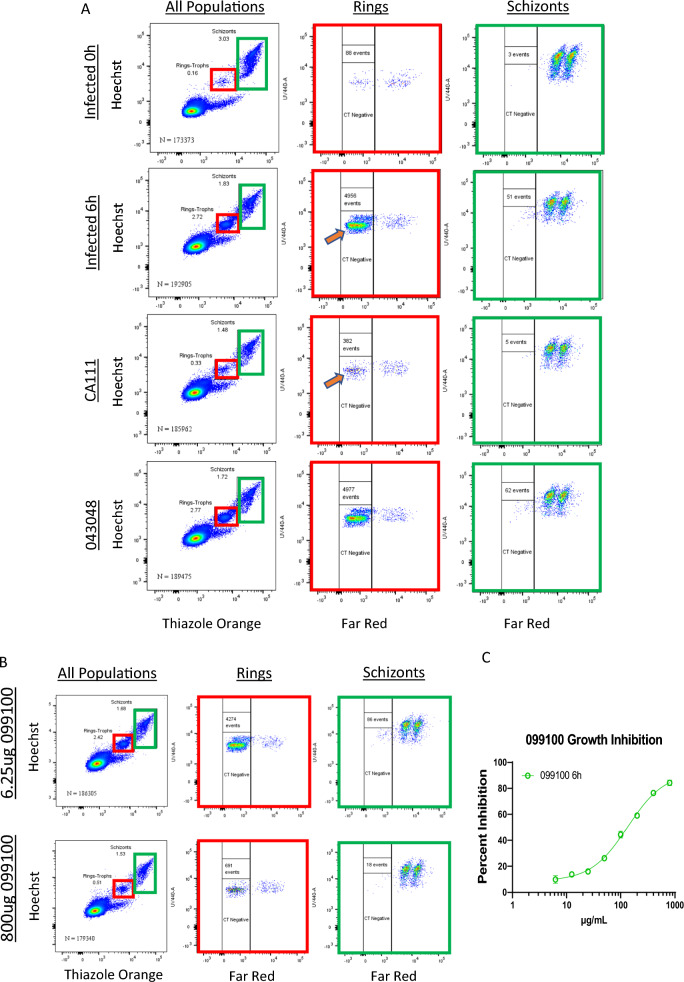
Experimental Design and Invasion inhibition activity for humAb 099100 to PvDBPII: Cultures were initiated from a cryopreserved stock of *P. knowlesi* A1.H.1 strain and were maintained in human RBCs at 2% haematocrit. Parasites were enriched for schizonts using a Nycodenz gradient. The schizont preparation was labelled with CellTrace to identify them as donor cells. Donor cells were then mixed with unlabelled recipient cells at a ratio of 1:20 and incubated with/without experimental reagents. **A** Rows one and two show cultures at time zero hour and at 6 h respectively, indicating new invasion events (orange arrow). After 6 h of culture, samples were stained with Hoechst 33342 for DNA content (Y-axis, and thiazole orange, left-hand panel and Far Red (CellTrace, middle and right panels) to identify rings (red boxes) that represent new invasion events (CT negative, N = 4956 at 6 h) and schizonts (green boxes). Row three shows the blocking of PkPvDBPOR invasion with a camelid nanobody CA111. Row four shows culture containing a negative control humAb 043048 (tetanus toxoid-specific). **B** The top and bottom rows show 6 h cultures treated with humAb 099100 at the lowest and highest concentration, respectively, of a two-fold dose response. **C** The full growth inhibition curve of 099100

**Fig. 2:**
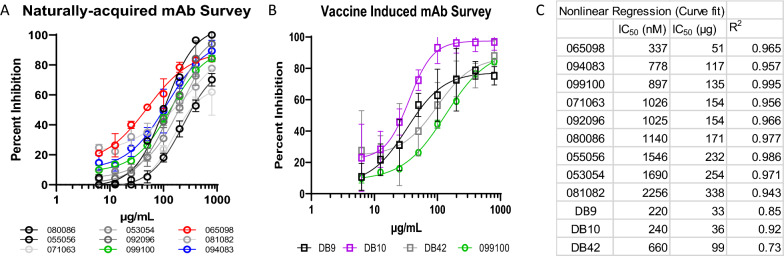
Invasion inhibition survey of all humAbs: **A** The full growth inhibition curves of 9 humAbs. **B** The full growth inhibition curves for 3 vaccine-induced humAbs; 099100 (green curve) overlaid for representative comparison to naturally-acquired humAbs, data referenced from panel A. **C** Nonlinear regression analysis to calculate IC_50_ values and goodness of fit (R^2^ values) for all mAbs tested. All percent inhibition points represent mean and standard deviation of triplicate cultures

After mixing the labelled donor cells with unlabelled recipient cells, measurement of samples at baseline (time zero) revealed up to 0.16% ring-stage parasites and 3.03% schizonts (Fig. 1A, upper panels). Greater than 99.9% of schizonts were labelled with CellTrace, whereas 88 events of ring-stage parasites were CellTrace negative (Fig. 1A, upper middle and right columns). The CellTrace-negative cells with ring-stage parasites likely represent early infection of newly added recipient target cells. The number of newly infected cells that are CellTrace negative markedly increased 55-fold (4956 events) by 6 h (Fig. 1A, second row) in the absence of antibodies or with a non-PvDBP-specific control humAb 043048 (tetanus toxin C-terminal fragment-specific humAb; Fig. 1A, lower row). The ring-stage parasite observed at time zero are subtracted from new invasion events in the 6-h cultures in different experimental conditions. To demonstrate that PkPvDBPOR invasion of human red cells is Duffy dependent, the nanobody CA111 (that recognizes an epitope on DARC to which PvDBPII binds) inhibited PkPvDBPOR invasion by 91% (Fig. 1A). The NA PvDBPII humAb 099100 is a focal point for comparisons in the PkPvDBPOR model as it was found to have the highest avidity in earlier studies and shown to consistently inhibit *P. vivax* clinical isolates in vitro [33, 44]. As shown in Fig. 1B and C, 099100 inhibited PkPvDBPOR erythrocyte invasion in a dose-dependent fashion up to 85% and showed an IC_50_ of 135 µg/mL. In addition to 099100, eight additional NA humAbs were tested and analysed for growth inhibition of PkPvDBPOR (Fig. 2A). Their IC_50_ values summarized in a table ranging from 51 µg/mL to 338 µg/mL, with R^2^ values above 0.94 (Fig. 2C). The NA humAb 065098 best inhibited PkPvDBPOR transgenic parasites with an IC_50_ of 51 µg/mL.

The VI humAbs, derived from humans vaccinated with the Sal 1 PvDBPII vaccine formulation [38], were evaluated on their ability to inhibit erythrocyte invasion and growth of PkPvDBPOR. Results for the independent tests of these three VI humAbs, DB9, DB10, and DB42, are summarized in Fig. 2B. These VI humAbs were characterized by IC_50_ values of 33 µg/mL, 36 µg/mL and 99 µg/mL for DB9, DB10, and DB42, respectively. Despite having lower IC_50_ values than 099100, DB9 (targets PvDBPII SD3 epitope [38]) and DB42 (PvDBPII subdomain epitope unknown) performed comparably to 099100. In contrast, the IC_50_ for DB10 was fourfold lower than 099100 and nearly reached 100% invasion inhibition at 100 µg/mL (Fig. 2B).

### HumAb combinations in the PkPvDBPOR GIA

Next, it was investigated whether combining two humAbs may have synergistic effects. The focus was on three humAbs, 099100, 094083, and 065098, based on distinct inhibition curves and different predicted PvDBPII binding epitopes from previously performed competition experiments [33]. HumAb 065098 demonstrated the most potent GIA effect of the NA antibodies. The NA humAbs 092096 and 053054 targets the PvDBPII-DARC binding interface in SD2 of PvDBPII assessed by X-ray crystallographic studies [14, 15] and exhibited competitive binding with 099100. This suggests that 099100 may bind to the same or nearby epitope [33], although this does not exclude the possibility that they recognized different but overlapping epitopes. Based on an absence of competitive binding with other humAbs, 094083 appeared to bind to a unique epitope [33]. These combination studies were also performed with all three VI humAbs.

**Fig. 3:**
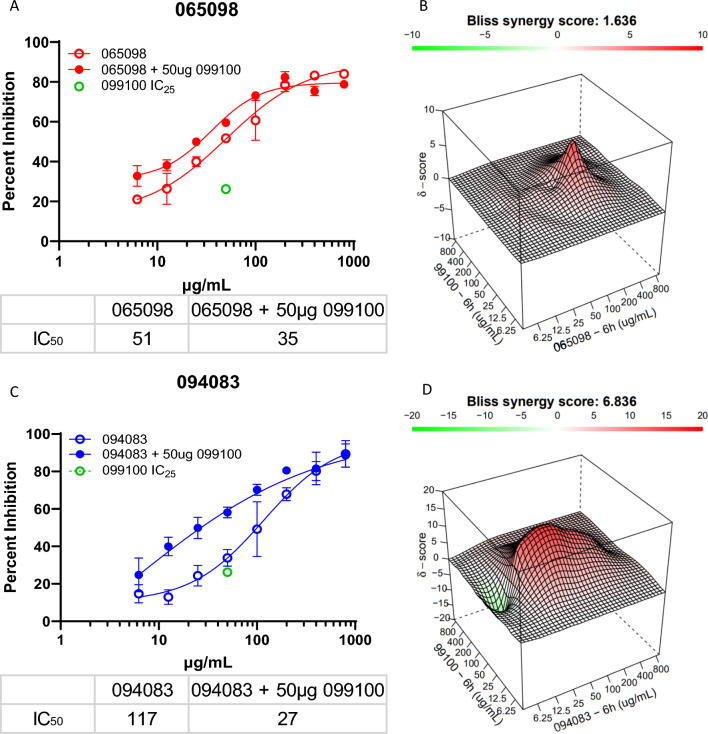
HumAb Synergy Experiments: **A**, **C** The first column shows growth inhibition curves for humAbs 065098, and 094083 individually, with and without the presence of 099100; 099100 was held constant at its IC_25_ (50 µg/mL) at each concentration (i.e., 800 µg/mL 065098 + 50 µg/mL 099100, 400 µg/mL 065098 + 50 µg/mL 099100, etc.); the green dot depicts the percent inhibition of 099100 at its IC_25_ (50 µg/mL); All points are mean and standard deviation of triplicate culture. The table below the figures shows differences in IC_50_ of 065098 and 094083 with and without 099100. **B**, **D** Assessment by SynergyFinder web-based application software to visualize multi-antibody multi dose combination data based on the independent model Bliss: 3-dimensional representation of the dose–response matrices showing varying concentrations of 065098 **B** and 094083 **D** on the x-axis, varying concentrations of 099100 on the y-axis (using data from Fig. 2), and synergy score (δ) on the z-axis; each matrix is colour coded to show synergy distribution, a corresponding Bliss synergy score (BSS), and topography (peaks/valleys) at specific concentrations; green/valleys denotes antagonism, red/peaks denotes additivity or synergy. Scale for BSS: score < 0 = antagonism, 10 < score > 0 = additivity, score > 9.9 = synergy

In these humAb combination studies, GIAs were performed with and without 099100 at its observed IC_25_ (50 µg/mL). Concentrations of the paired humAbs (065098 and 094083) were diluted two-fold, starting at 800 µg/mL down to 6.25 µg/mL (Fig. 3A, C). Both 065098 and 094083 display increased levels of inhibition when combined with 099100, particularly 094083 (Fig. 3). The humAb 065098 showed marginal additivity (BSS of 1.64), peaking at 100 µg/mL (Fig. 3B). The combination of 094083 and 099100 exhibited stronger additivity (BSS of 6.84), and an expanded peak from approximately 12.5 µg/mL to 200 µg/mL, suggesting additivity at a broad range of concentrations (Fig. 3D).

**Fig. 4:**
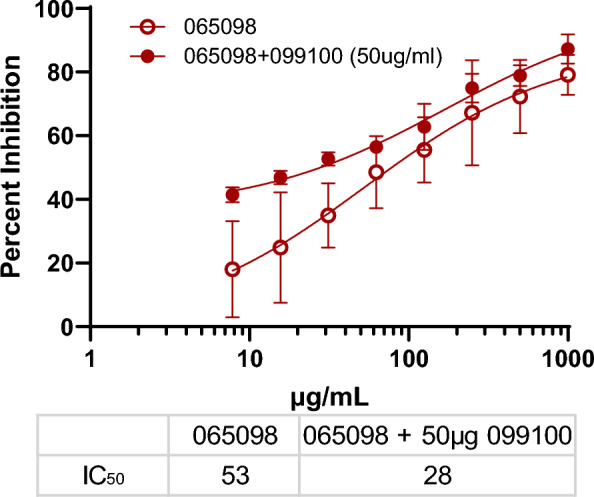
Comparative Growth Inhibition Assay of *P. vivax*: Growth inhibition curves for humAbs 065098, with and without the presence of 099100; 099100 was held constant (50 µg/mL) at each concentration. All points are mean and standard deviation based on 3 biological replicates of separate clinical *P. vivax* isolates. Each *P. vivax* isolate was performed in triplicate at each concentration

To validate whether invasion inhibition observed with different humAbs using the PkPvDBPOR model represents that observed with *P. vivax*, the interactions of humAbs 065098 and 099100, described in Fig. 3A with PkPvDBPOR, were examined using clinical isolates of *P. vivax* (Fig. 4). The blocking potential of humAb 065098 with Pv had IC_50_ of 53 µg/mL that closely resembled that observed with PkPvDBPOR with an IC_50_ of 50 µg/mL. The addition of 50 µg/mL of 099100 had an additive effect to the dose–response curve of 065098 that reduced the IC_50_ to 28 µg/mL. The same combination of humAbs using PkPvDBPOR showed an IC_50_ of 35 µg/mL (Fig. 3A). Thus, the PkPvDBPOR system closely recapitulates the effects of humAbs on inhibiting *P. vivax* invasion of reticulocytes.

**Fig. 5:**
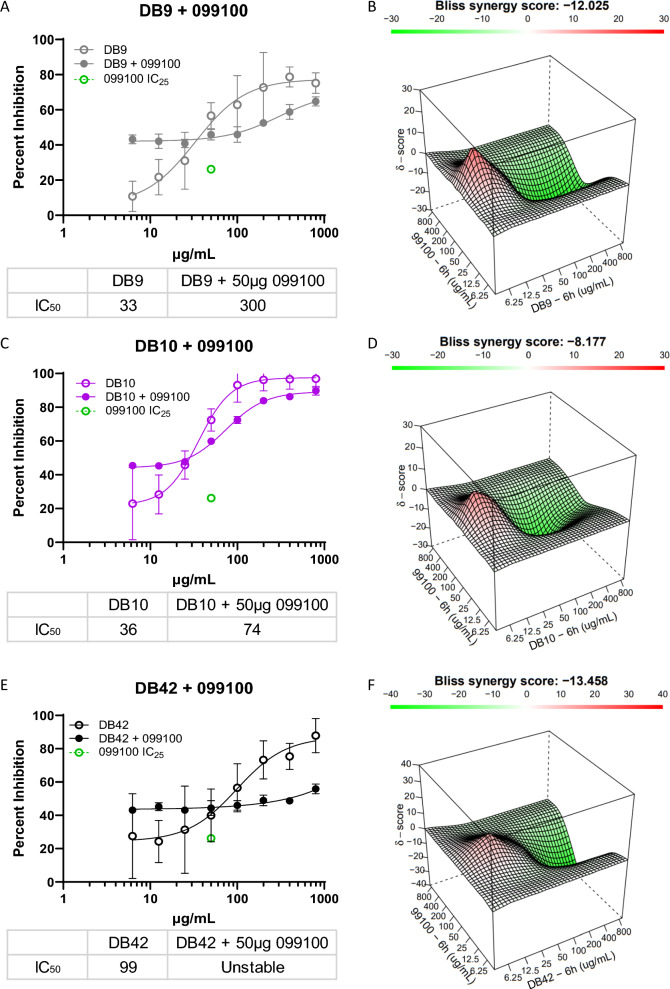
Invasion inhibition with combination of humAb 099100 with humAbs DB9, DB10 and DB42: **A**, **C**, **E** Growth inhibition curves for humAbs DB9, DB10, DB42, and 099100 individually, with and without the presence of 099100 held constant at its IC_25_ (50 µg/mL, green dot). All points represent mean and standard deviation with triplicate cultures. **B**, **D**, **F** 3-dimensional representations of the dose–response matrices showing varying concentrations of DB9 (**B**), DB10 (**D**), and DB42 (**F**) on the x-axis, respectively from top to bottom, 099100 on the y-axis, and synergy score (δ) on the z-axis. Refer to legend in Fig. 3 describing Bliss synergy matrix and scores

To evaluate whether a combination of humAbs generated by exposure to the PvDBPII or vaccination might exhibit synergistic effects, individual assessments were performed of DB9 (of note DB9 is predicted to bind PvDBPII SD3), DB10 and DB42 (Fig. 5A, C and E), in combination with 099100. Relative to their individual inhibition curves, these humAbs produced antagonistic effects (Fig. 5B, D and F) when the concentration of monoclonals exceeded 25 µg/mL. The BSS for each combination was − 12.03 (DB9 + 099100), − 8.18 (DB10 + 099100), and − 13.5 (DB42 + 099100). At lower concentrations of the VI humAbs, they showed additive effects; IC_50_ values for DB9 and DB10 in combination with a fixed amount of 099100 had increased to 300 µg/mL and 74 µg/mL, respectively (Fig. 5A and C). The values generated in the DB42 + 099100 combination did not generate a distinct inhibition curve, therefore, a stable IC_50_ could not be calculated for DB42 (Fig. 5E).

**Fig. 6:**
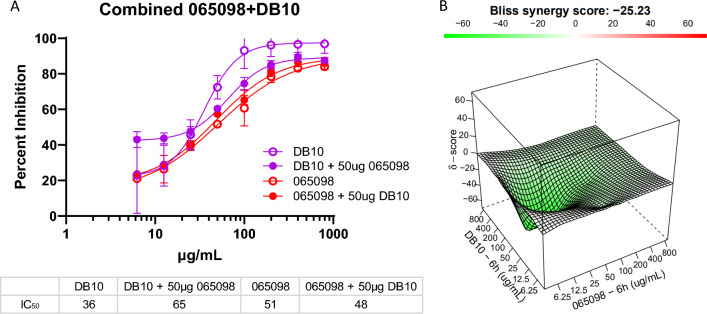
Combination experiment with humAbs 065098 and DB10: **A** Growth inhibition curves for humAbs DB10 and 065098 individually and in combination. One antibody was varied in a two-fold dose response at eight concentrations while the other was held constant at 50 µg/mL, and then inversely (red and violet curves); individual curves for DB10 (dashed blue curve) and 065098 (dashed green curve) are overlaid for comparison, data referenced from Figs. 2 and 3. All points represent mean and standard deviation with triplicate cultures. **B** 3-D dose–response matrices showing the concentrations of 065098 on the x-axis, DB10 on the y-axis, and synergy score (δ) on the z-axis. Refer to legend in Fig. 3 describing Bliss synergy matrix and scores

The best blocking humAbs, NA 065098 and VI DB10, were examined in combination as to whether they would generate a synergistic response (Fig. 6). The combination experiments were performed reciprocally, with one antibody being varied across a two-fold dose–response range (eight concentrations—800 µg/mL to 6.25 µg/mL) with the other being fixed at 50 µg/mL. These combination experiments had no additive or synergistic effect (Fig. 6A), as inhibition values were only marginally different from the independent inhibition dose responses. IC_50_ values for DB10 + 065098 (fixed concentration) increased from 36 µg/mL to 65 µg/mL and decreased from 51 µg/mL to 48 µg/mL for the 065098 + DB10 (fixed concentration). According to the synergy distribution and tensor, there was a strong antagonism between the two antibodies, resulting in a BSS of -25.2 (Fig. 6B).

### Avidity of vaccine-induced mAbs

**Fig. 7:**
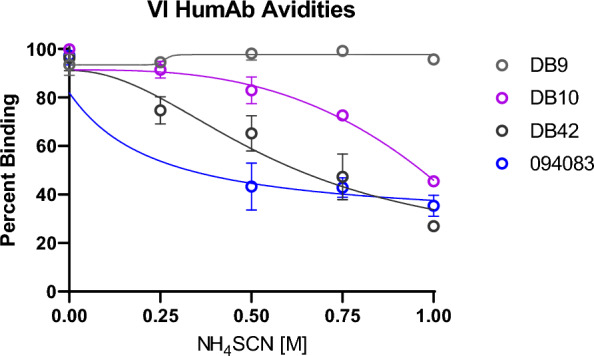
Avidity of VI humAbs: X-axis shows the concentrations of chaotropic agent ammonium thiocyanate (NH_4_SCN). Y-axis shows percent binding of humAbs DB9 (grey), DB10 (violet), DB42 (black), and 094083 (blue) to recombinant protein PvDBPII following exposure to NH_4_SCN at different concentrations

Differences in antibody avidities may account for potential additive or antagonistic interactions between VI and NA human mAbs. Antibody avidity assays were performed for VI mAbs DB9, DB10 and DB42 using identical methods for NA humAbs reported previously (Fig. 7) [33]. Regarding previous studies, NA mAb 094083 was included, which precisely recapitulated percent binding follow treatment with chaotropic agent NH_4_SCN at 0.5 M and 1.0 M to that observed previously [33]. DB9 had highest avidity, followed by DB10 and DB42. Including the results from previous studies, 099100 and DB9 showed the highest avidity of NA and VI humAbs tested [33].

## Discussion

This study used PvDBPII-specific humAbs from naturally infected and vaccine-exposed people [21, 22] to inhibit human erythrocyte invasion by *P. knowlesi* genetically modified to express the Sal I allele of PvDBP, PkPvDBPOR [32–34]. The in vitro studies demonstrate an efficient approach for evaluating these antibodies individually and in combination. The invasion inhibition of these humAbs shows using the PkPvDBPOR is recapitulated using clinical isolates of *P. vivax*. This easily cultured parasite line may help optimize the development of humAb prophylaxis and treatment strategies against blood-stage *P. vivax* malaria.

Recent studies characterized these PvDBP-specific humAbs in the context of binding inhibition analyses [33, 38], in vitro inhibition (ex vivo* P. vivax* and in vitro* P. knowlesi*) [33, 38] and X-ray crystallography illustrating epitope recognition by some humAbs [27, 38]. Here, the PkPvDBPOR in vitro system is used to perform individual invasion inhibition studies in side-by-side comparisons of the NA and VI PvDBP-specific humAbs. To test the potential for additive, synergistic, or antagonistic effects, humAbs with known affinity and binding site characteristics were studied to test hypotheses regarding specific interactions between these PvDBPII-specific humAbs (and the target antigen).

The examination of different combinations of mAbs PvDBPII is important for its clinical development for malaria prophylaxis or treatment, as targeting two distinct epitopes could lead to improved protection against malaria as has been seen with viral infections like HIV-1, Ebola, and SARS-CoV-2 [47–49]. In tests on humAbs individually, the IC_50_ values observed for NA humAbs ranged from 51 to 338 µg/mL (340–2250 nM). In comparison, the vaccine-induced humAbs were three of the four best inhibitors with IC_50_ values of 33, 36, and 99 µg/mL (220, 238, and 660 nM) for DB9, DB10, and DB42 respectively (Mann Whitney P-value: 0.0182); the IC_50_ for the DARC-specific camelid nanobody, CA111, was 0.25 µg/mL (17 nM). The higher efficacy of the VI humAbs is not surprising, considering they were generated from individuals immunized with Sal I PvDBP and were tested against PkPvDBPOR containing the Sal I variant [38]. By contrast, the NA humAbs were generated from Cambodian donors [33]. In Cambodia, the Sal I variant of PvDBP is present but is not as common, potentially introducing more epitope variation by the naturally acquired humAbs [23]. The humAbs reached a maximum inhibition above 80% at 800 µg/mL (5.3 µM); 80% inhibition for CA111 was 2 µg/mL (133 nM). The binding avidity data did not necessarily correspond with the erythrocyte invasion inhibition data for PkPvDBPOR (e.g., 065098 and 094083 exhibited among the weaker avidities but had the strongest erythrocyte invasion inhibitory effects, based on lowest IC_50_ blocking PkPvDBPOR in vitro invasion) [33]. This suggests humAb access to an epitope that better spans critical binding residues for the DARC:DBPII interaction is more important than avidity for humAb's ability to inhibit *P. vivax* invasion into reticulocytes.

The humAb combinatorial tests begin to identify specific humAb partners that may be able to optimize the therapeutic use of these reagents. In earlier studies, humAb 099100 is well characterized as having the greatest avidity and demonstrated invasion inhibition in short-term *P. vivax* ex vivo assays of Brazilian and Cambodian isolates, informing the decision to select it as the constant humAb throughout the first set of combination experiments [33]. Additionally, 099100 binding to PvDBP was competitively inhibited by 065098, but not by 094083—suggesting that these two humAbs bind to different epitopes. The negligible additivity by 065098 + 099100 is attributed to be competitive binding for the same position. The potential for synergy was more significant for humAb 094083, for having the second lowest IC_50_ value (117 µg/mL), and its linear epitope that does not overlap with the epitope of 099100. Combining 094083 + 099100 resulted in significantly more inhibition and a strong additive signal (BSS of 6.84), indicating noncompetitive inhibition. Following this logic, it was hypothesised that combinations of PvDBPII SD2-binding humAbs with PvDBPII SD3-binding humAbs would demonstrate even greater additive, possibly synergistic inhibition.

**Fig. 8:**
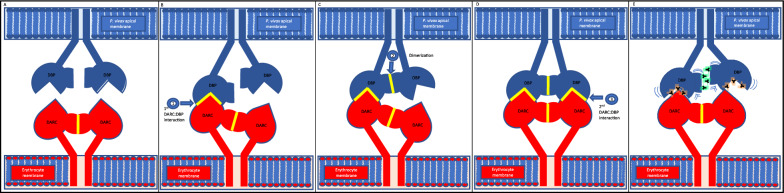
Binding organization of the DBP-DARC heterotetramer: **A** Uncoupled DBP monomers on apical membrane of *P. vivax* and the DARC dimer on the surface of the erythrocyte membrane. **B**–**D** Multi-step binding sequence of DBP to DARC – initial binding of DBP monomer to the DARC dimer (1), dimerization of DBP monomers (2), and secondary binding of DBP dimer to DARC dimer. **E** Interference of heterotetramer formation by SD2-binding (orange) and SD3-binding (green) humAbs

PvDBPII interaction with DARC is a multi-step interaction to form a heterotetramer (Fig. 8) [14, 15]. The first interaction is the formation of PvDBPII monomer where SD2 of PvDBPII interacts with DARC. Thus, humAbs that inhibit this step might be expected to be more potent, especially if they have high avidity or affinity. HumAbs that target SD3, such as DB9 [38], that prevent step 2 formation of the dimer that requires SD3 could also be effective. The hypothesis was that humAbs that target both steps might be synergistic, but this did not occur. Indeed humAb 099100, with a similar avidity to DB9 interfered with DB9 inhibition at higher concentrations. One interpretation is that the high avidity but less effective blocking of mAb 099100, inhibited mAb DB9 access to the SD3 dimer interface, evident only in higher concentrations when 099100 saturates available epitopes. A second possibility is that complexing of 099100 to PvDBPII results in a conformational change that reduces humAb DB9 binding affinity. DB10 and DB42 was combined with 099100 individually, resulting in antagonism in both cases. Where DB10 and DB42 bind to PvDBPII is unknown but may have a similar mechanism of antagonism as observed with DBP9. Combination experiments with the best NA 065098 and VI DB10 humAbs, showed a difference between which antibody was varied in concentration or tested as a single concentration. If 065098 is varied in the combination experiment while DB10 is held constant, there is no appreciable difference in the inhibitory dose–response curve or IC_50_ values. However, if DB10 is varied while 065098 is held constant in the combination experiment, the curve displays additive inhibition at lower concentrations but antagonism at higher concentrations. Previous research has shown that 065098 and 099100 compete with another humAb, 053054, which also has a binding epitope in the functional region of DBP, the dimer interface on SD2 [33]. The mechanism for antagonism might also be similar to that described for 099100 and DB9. This data is significant in light of *P. vivax* blood stage vaccines being focused on merozoite invasion proteins, specifically DBP [34, 50]. The antagonism identified throughout the combination experiments could reveal a limitation in developing strain-transcending antibodies that are specific to DBP and may contribute to the only 50% reduction in parasite growth during a controlled human malaria infection observed in a recent phase 1/2a clinical trial following vaccination with rDBPII [51].

The independent inhibitory performances of 065098, 094083, DB9, DB10, and DB42 highlight 5 potential protective antibodies and epitopes that could be the target of future immunotherapies. Indeed, the concentration at maximum inhibition for the majority of the humAbs is 800 µg/mL; too high to apply in a clinical setting, assuming the PkPvDBPOR in vitro system directly translates to an in vivo model. Preliminary data from an ex vivo GIA of 065098 against clinical *P. vivax* isolates supports that PkPvDBPOR is a good model for the translational study of humAbs against *P. vivax* antigens. The ex vivo GIA reports similar IC_50_ curves and values for 065098 in *P. vivax* (53 µg/mL) and *P. knowlesi* (51 µg/mL) (Fig. 4). Although, these IC_50_ values are 2 to fivefold higher than *P. falciparum* monoclonal studies, such as *P. falciparum* circumsporozoite protein studies, that show protection in vivo mice [52, 53] and humans [54, 55]. Ideally, high-efficacy monoclonals specific to antigens in the pre-erythrocytic infection would work in concert with humAbs targeting blood stage invasion ligands like PvDBP. The more potent pre-erythrocytic antibodies would work to neutralize sporozoites before they can infect hepatocytes, and breakthrough infections would be ablated by the less potent humAbs detailed here.

## Conclusion

Using monoclonal antibodies against viral and parasite invasion ligands has demonstrated how challenging the discovery of the optimal combination of reagents to block infection can be [56–62]. The technologies applied in this and recent studies [33, 38] appear to expand the generation and evaluation of humAb therapeutic reagents for treatment and prophylaxis of *P. vivax* malaria that have not been possible previously because of the difficulties of in vitro methods for the culture of *P. vivax*. The treatment of *P. vivax* malaria in permissive non-human primate models is an essential next step for evaluating these potentially protective humAbs. For example, the humAbs may have greater in vivo activity enhanced by Fc-mediated activity. The further development of these methods and reagents will be important if *P. vivax* is to be eliminated as a significant global public health challenge.

## Data Availability

Data used during the current study are available from the corresponding author upon reasonable request.

## Abbreviations

ACKR1: Atypical chemokine receptor 1
DARC: Duffy antigen receptor for chemokines
DBP: Duffy binding protein
DBPII: Cysteine-rich region II of Duffy binding protein
Fy: Duffy
GIA: Growth inhibition assay
HumAbs: Human monoclonal antibodies
NA: Naturally acquired
P: *Plasmodium*
PkCM: *P. knowlesi* Culture media
PkPvDBPOR: Genetically modified Pk strain A1-H.1 PvDBP OR /Δ14
RBC: Red blood cells
Sal I: Salvador I strain
SD: Subdomain
VI: Vaccine-induced
